# Longitudinal changes in blood-borne geroscience biomarkers: results from a population-based study

**DOI:** 10.1007/s11357-025-01666-x

**Published:** 2025-04-24

**Authors:** Anna Picca, Ngoc Viet Nguyen, Riccardo Calvani, Matilda Dale, Claudia Fredolini, Emanuele Marzetti, Amaia Calderón-Larrañaga, Davide Liborio Vetrano

**Affiliations:** 1 Department of Medicine and Surgery, LUM University, Casamassima, Italy; 2 Fondazione Policlinico Universitario “Agostino Gemelli” IRCCS, Rome, Italy; 3 Clinical Epidemiology Division, Department of Medicine Solna, Karolinska Institutet, Stockholm, Sweden; 4 Department of Geriatrics, Orthopedics and Rheumatology, Università Cattolica del Sacro Cuore, Rome, Italy; 5 Affinity Proteomics Stockholm, Science for Life Laboratory, Department of Protein Science, School of Engineering Sciences in Chemistry, Biotechnology and Health (CBH), Royal Institute of Technology (KTH), Solna, Sweden; 6 Aging Research Center, Department of Neurobiology, Care Sciences and Society, Karolinska Institutet and Stockholm University, Stockholm, Sweden; 7 Stockholm Gerontology Research Center, Stockholm, Sweden

**Keywords:** Inflammation, Metabolism, Multi-marker, Neurodegeneration, Organ dysfunction, Senescence

## Abstract

Multi-marker approaches are well suited for untangling the intrinsic complexity of aging and related conditions. Herein, we quantified (1) baseline concentrations of a panel of geroscience biomarkers pertaining to four biological domains (i.e., metabolism, inflammation, vascular/organ dysfunction and cellular senescence, and neurodegeneration) in individuals aged ≥60 years; (2) investigated linear and non-linear changes in biomarker levels over a 6-year period according to age and sex; and (3) described the relationships among geroscience biomarkers at baseline and follow-up. We found that repeated measures of age-dependent changes of 47 blood-borne biomarkers over 6 years had differential associations depending on the biological domains. The most relevant biomolecules in the associations between age and repeated assessments were (1) adiponectin, C-peptide, renin (metabolism), (2) CXCL10, IL-1α, IL-1β, IL-6, IL-10, IL-12p70, MPO (inflammation), (3) cystatin C, MMP7, MMP12, VCAM-1 (vascular/organ dysfunction and cellular senescence), and (4) S100B and Tau protein (neurodegeneration). Among these molecules, a negative association with increasing age was found for IL-1α, IL-1β, IL-12p70, S100B, and Tau protein. Non-linear relationships were also identified with age for IGFBP-1, leptin, β2M, TNFRSF1B, fibrinogen, GDF-15, N-cadherin, and BDNF. Our results indicate that inflammatory and metabolic biomolecules are strongly associated with aging over 6 years of follow-up. Whether the biological pathways reflected by these biomarkers contribute to the aging process or are associated with negative health-related events needs to be explored through comprehensive multi-omics longitudinal analysis in larger cohorts.

**Supplementary Information:**

The online version contains supplementary material available at 10.1007/s11357-025-01666-x.

## Introduction

Aging has a complex, multifactorial, and heterogeneous biology that affects all bodily systems. A transition from “homeostatic symphony to cacophony” has been advocated by Fried et al. [1] to describe the bulk of events underlying the aging process and leading to negative health-related events. The geroscience paradigm proposes a set of processes and pathways as core mechanisms of aging and triggers of age-related conditions [2, 3]. These pathways, known as hallmarks of aging, were originally identified in genomic instability, telomere attrition, epigenetic alterations, mitochondrial dysfunction, loss of proteostasis, deregulated nutrient sensing, cellular senescence, stem cell exhaustion, and altered intercellular communication [3]. Subsequently, additional hallmarks have been proposed, which include compromised autophagy, splicing dysregulation, altered mechanical properties, microbiome disturbance, and inflammation [2]. The hallmarks of aging can therefore be interrogated to identify biological markers of aging for assessing the individual susceptibility to chronic diseases and loss of function [4].

Multi-marker analytical approaches are well suited for untangling the intrinsic complexity of aging and related conditions, such as frailty [5–7] and neurodegeneration [8–10]. The characterization of age- and sex-specific trajectories of geroscience markers is necessary to establish their actual value as metrics of biological aging.

In the present study, after a careful revision of the literature and based on the results of a recent systematic review and meta-analysis [11], a multi-marker immunoassay was used for the quantification of a multi-domain panel of blood-borne biomolecules. Molecules to be included in the biomarker panel were selected based on their belonging to one of the biological domains of interest and the availability of commercial kits for their measurement using analytical platforms available in-house. The objectives of the present investigation were to (1) quantify baseline concentrations of a panel of geroscience biomarkers in a well-characterized sample of individuals aged ≥ 60 years; (2) investigate linear and non-linear changes in biomarker levels over a 6-year period according to age and sex; and (3) describe the relationships among geroscience biomarkers at baseline and follow-up.

## Methods

### Study population and data collection

The present study was conducted in a sub-sample of participants enrolled in the Swedish National Study on Aging and Care in Kungsholmen (SNAC-K) [12]. SNAC-K is a community-based longitudinal study of randomly selected individuals aged ≥ 60 years living in the Kungsholmen district of Stockholm [12]. In the current analysis, a convenience sample of 234 individuals was randomly selected from the original SNAC-K population by age cohort-stratified sampling. A sample of 27 − 30 individuals from each of the seven younger cohorts (60 − 89 years) and a sample of 30 from the four remaining cohorts (≥ 90 years) were selected. We used data from the assessment at baseline and at year six of follow-up (i.e., the first follow-up assessment of participants < 78 years and the second follow-up assessment of those ≥ 78 years). Detailed protocol for data collection at each assessment can be found at https://www.snac-k.se/about/study-plan/. All waves of the SNAC-K study were approved by the Regional Ethical Review Board in Stockholm, Sweden (Dnrs: KI 01–114; 04–929/3). Written informed consent was obtained from all participants or next of kin. as appropriate, prior to enrolment.

### Blood collection and biomarker measurements

Non-fasting blood samples were obtained from all SNAC-K participants at each assessment, processed, and stored at − 80 °C until analysis. For the present study, 47 biomarkers pertaining to four physiological domains were quantified: 19 markers of inflammation, 13 markers of vascular/organ dysfunction and cellular senescence, nine metabolic markers, and six markers of neurodegeneration (Table 1). Biomarkers were quantified in serum samples at baseline and at year six of follow-up. Quantifications were carried out at the Affinity Proteomics-Stockholm Unit of the SciLifeLab (Solna, Sweden) using a custom designed Magnetic Luminex Assays − Human Premixed Multi-Analyte assay (Luminex Corporation, Austin, TX, USA). The average intra- and intercoefficient variations for replicated samples were 4.5% and 5.9%, respectively.

**Table 1:** Composition of the geroscience biomarker panel

**Physiological domain(s)**	**Biomarkers**
Inflammation	β2-microglobulin, C-C motif chemokine ligand (CCL) 2, 3, 4, and 11, C-reactive protein (CRP), interferon γ (IFN-γ), interleukin (IL)-1α, 1β, 2, 6, 8, 10, 12 p70, and 12, myeloperoxidase (MPO), tumor necrosis factor α (TNF-α), tumor necrosis factor receptor superfamily member 1B (TNFRSF1B), CXCL10
Metabolism	Adiponectin, connecting peptide (C-peptide), growth hormone (GH), insulin-like growth factor-binding protein (IGFBP) 1 and 3, insulin, leptin, prolactin, renin
Neurodegeneration	α-synuclein, brain-derived neurotrophic factor (BDNF), glial cell line-derived neurotrophic factor (GDNF), nerve growth factor β (NGF-β), S100 calcium-binding protein B (S100B), Tau protein
Vascular/organ dysfunction and cellular senescence	Cystatin C, epidermal growth factor (EGF), ephrin type-A receptor 2 (EphA2), E-selectin, fibronectin, growth differentiation factor 15 (GDF-15), intercellular adhesion molecule 1 (ICAM-1), matrix metalloproteinase (MMP) 7 and 12, N-cadherin, P-selectin, vascular cell adhesion molecule 1 (VCAM-1), vascular endothelial growth factor (VEGF)

### Other variables

Participant characteristics were obtained from comprehensive clinical and functional assessments performed by trained physicians, nurses, and neuropsychologists at each wave of SNAC-K assessment. Socio-demographic information (e.g., age, sex, education) was collected through nurse interviews. The number of medications used at the time of assessment was recorded based on self-reported information. Diagnoses of chronic diseases were identified through self-reports, medical examination, laboratory tests, medical charts, medications, and data from the Swedish National Patient Register (inpatient and outpatient specialist care). In the current analysis, for descriptive purposes, we included the following conditions: hypertension, ischemic heart disease, heart failure, atrial fibrillation, depression and mood disorders, cerebrovascular disease, chronic obstructive pulmonary disease (COPD), asthma, cancer, and chronic kidney disease. Cognitive function was assessed by physicians through the Mini-Mental State Examination (MMSE). Physical function was examined using the usual gait speed (m/s) measured on a 6-m path or 2.44-m path if participants self-reported a slow or very slow walking speed.

### Statistical analysis

Baseline characteristics of participants were reported by means of descriptive analyses. Outliers in biomarker levels were defined as data points that lied beyond 1.5 times the interquartile range above or below the upper lower quartile, respectively (Tukey’s fence method). Among identified outliers, implausible values were removed from the analysis (e.g., MPO levels of > 1000 μg/L, NGF-β levels of > 50 pg/mL).

#### Characterization of changes in biomarker levels over time

The distribution of biomarker levels at baseline and follow-up was visualized using density plots and compared by paired *t*-tests. Biomarker concentrations were then standardized by re-scaling based on their distribution at baseline in the study sample. Baseline mean and standard deviation values of biomarker concentrations were, therefore, transformed into 0 and 1, respectively. The means of standardized concentrations at baseline and follow-up were plotted to visualize the pattern of changes from baseline to year six of individual biomarkers. In a sensitivity analysis, the standardization was carried out using baseline measures of participants 60 − 69 years (i.e., the youngest group) as the reference group.

#### Assessment of the association of age and sex with longitudinal repeated measures of blood biomarkers

Linear mixed-effects models were used to examine the association between age, sex, and biomarker concentrations measured twice over 6 years. A random effect was added at the individual level. Two models were built. In model 1, we included age at measurement and sex as the independent variables. In model 2, a quadratic term of age was added to allow for the study of non-linear patterns. We reported the coefficients and their 95% confidence intervals (CIs) of model 1. If the coefficient for the quadratic term of age was statistically significant, the outputs from model 2 were reported additionally.

#### Cross-sectional correlations of biomarkers and cluster analysis

For each measurement (baseline and follow-up), correlations between pairs of biomarkers were assessed using Pearson’s correlation analysis. A correlation matrix of 47 biomarkers was generated. Hierarchical clustering was then applied considering similarity measures (e.g., correlation distance) between each biomarker pair. A dendrogram depicting the correlation distance between biomarkers was generated to visualize groups of biomarkers that were more strongly correlated than others. The dendrogram was then combined with a heatmap of the correlation matrix to illustrate the clusters of biomarkers and the strength of correlation between biomarkers, respectively. We also calculated the ratio between the correlation coefficients at follow-up and at baseline for each pair of biomarkers to examine changes in correlations over time.

All analyses were performed using R (Version 4.1.2). Two-sided tests were used with a significance level of 5%.

## Results

### Participant characteristics

Baseline characteristics of the 234 participants included in the analysis are reported in Table 2. As compared with SNAC-K participants not included in this analysis, study participants were older (77.6 vs. 74.5 years), less educated (20.5% vs. 17.5% reported primary school education), had a higher MMSE score (28.5 vs. 27.0), and had a higher prevalence of chronic kidney disease (45.7% vs. 32.4%; *p*< 0.05 for all).

**Table 2:** Baseline characteristics of the study participants according to sex

**Characteristics**	**Men *n* = 72 (31%)**	**Women *n* = 162 (69%)**	***p* value**
Age (years), mean (SD)	76.5 (10.8)	78.1 (9.8)	0.264
Primary school education, *n* (%)	11 (15.3)	37 (22.8)	0.065
Number of diseases, mean (SD)	3.8 (2.2)	4.3 (2.2)	0.125
Number of medications, mean (SD)	3.1 (3.0)	4.4 (3.3)	0.008
Hypertension, *n* (%)	55 (76.4)	127 (78.4)	0.733
Ischemic heart disease, *n* (%)	15 (20.8)	24 (14.8)	0.254
Heart failure, *n* (%)	7 (9.7)	13 (8.0)	0.668
Atrial fibrillation, *n* (%)	6 (8.3)	16 (9.9)	0.709
Depression and mood disorders, *n* (%)	5 (6.9)	15 (9.3)	0.559
Cerebrovascular disease, *n* (%)	4 (5.6)	9 (5.6)	0.999
COPD/asthma, *n* (%)	6 (8.3)	13 (8.0)	0.936
Cancer, *n* (%)	8 (11.1)	12 (7.4)	0.350
Chronic kidney disease, *n* (%)	21 (29.2)	86 (53.1)	0.001
Mini Mental State Examination score, mean (SD)	28.7 (1.3)	28.5 (1.8)	0.288
Gait speed (m/s), mean (SD)	1.1 (0.4)	1.0 (0.4)	0.172
Hospitalization in previous year, *n* (%)	12 (16.7)	23 (14.2)	0.625

*SD* standard deviation, *COPD* chronic obstructive pulmonary disease

Among study participants, women reported a greater number of medications (4.4 vs. 3.1) and had higher prevalence of chronic kidney disease (53.1% vs. 29.2%) than men. None of the other sociodemographic, clinical, or functional characteristics differed significantly between sexes.

### Distribution of biomarker levels at baseline and follow-up

**Fig. 1:**
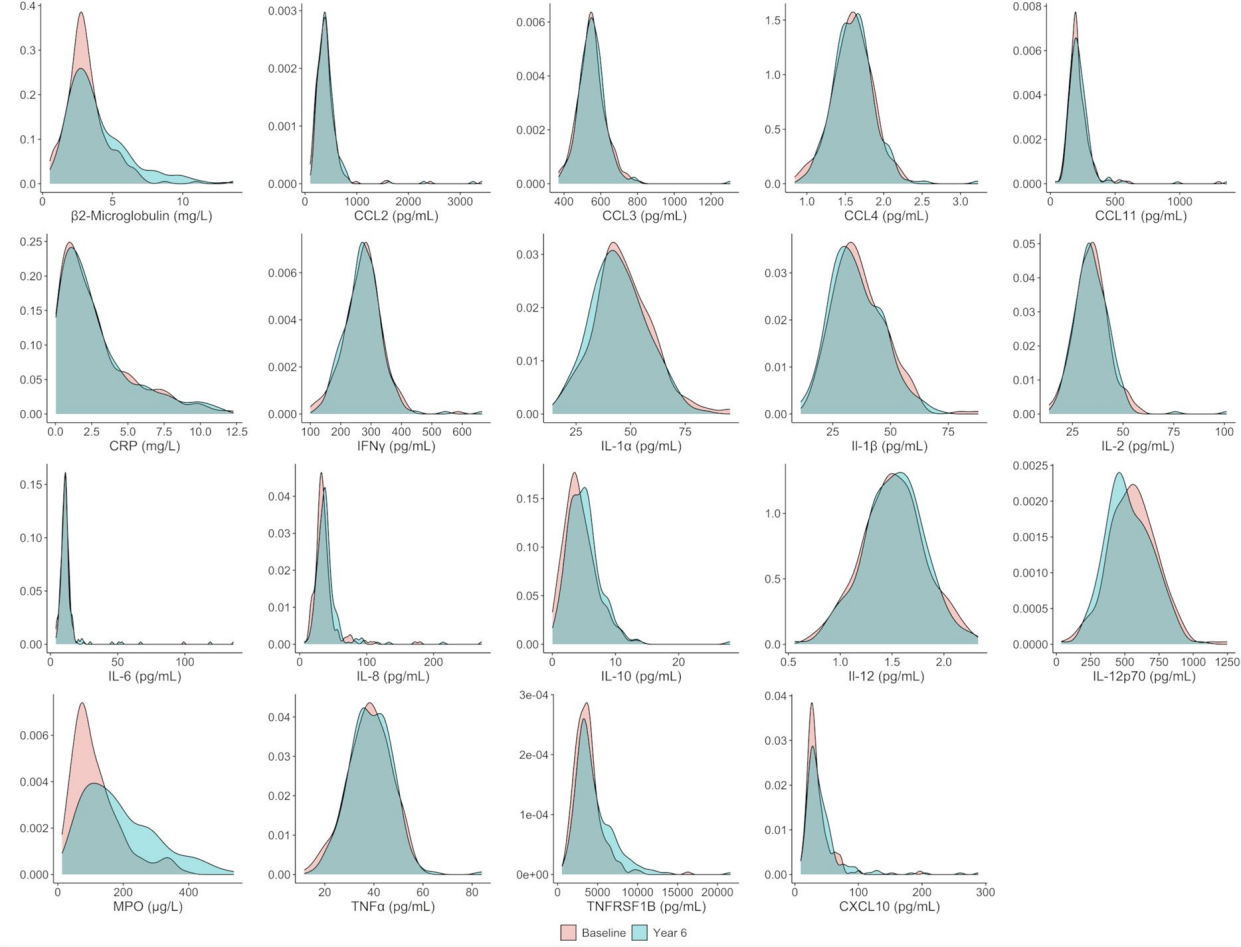
Density plots describing the distribution of inflammatory biomarker levels at baseline and at year six of follow-up. Abbreviations: CCL, C-C motif chemokine ligand; CRP, C-reactive protein; CXCL10, C-X-C motif chemokine ligand 10; IFN-γ, interferon gamma; IL, interleukin; MPO, myeloperoxidase; TNF-α, tumor necrosis factor α; TNFRSF1B, tumor necrosis factor receptor superfamily member 1B; VCAM-1

**Fig. 2:**
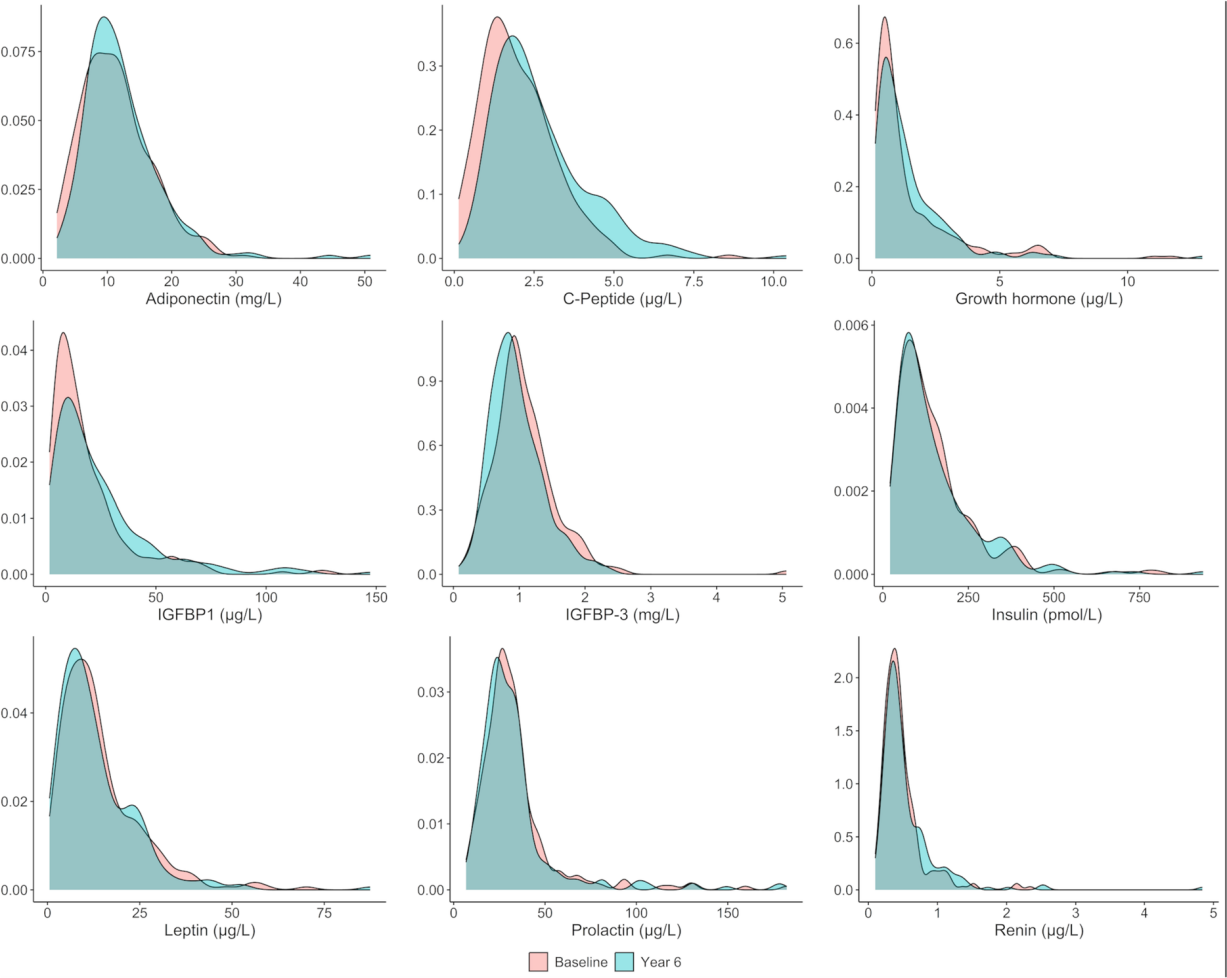
Density plots describing the distribution of metabolic biomarker levels at baseline and at year six of follow-up. Abbreviations: C-Peptide, connecting peptide; IGFBP, insulin-like growth factor-binding protein

**Fig. 3:**
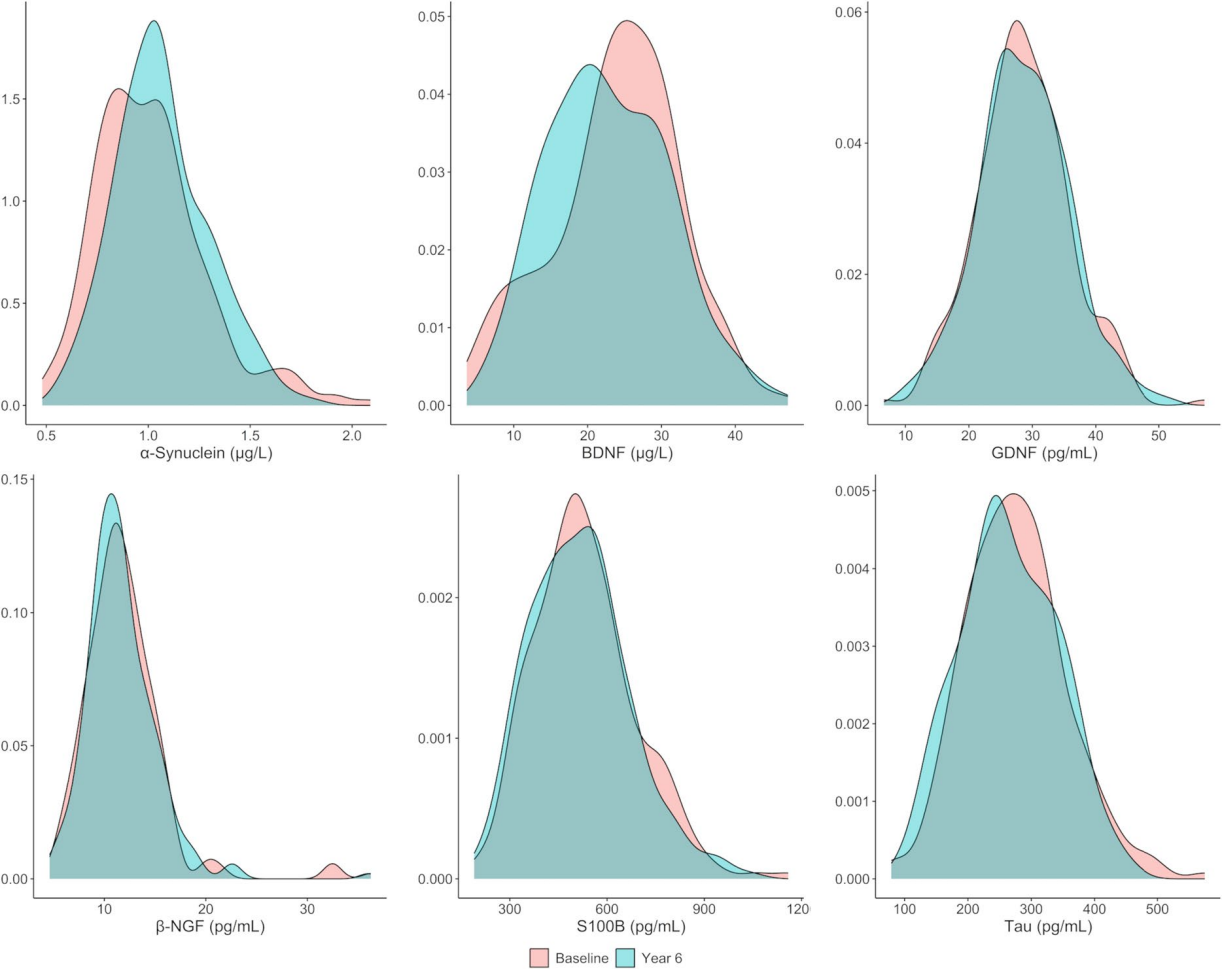
Density plots describing the distribution of neurodegeneration biomarker levels at baseline and at year six of follow-up. Abbreviations: BDNF, brain-derived neurotrophic factor; β-NGF, nerve growth factor β; GDNF, glial cell line-derived neurotrophic factor; S100B, S100 calcium-binding protein B

**Fig. 4:**
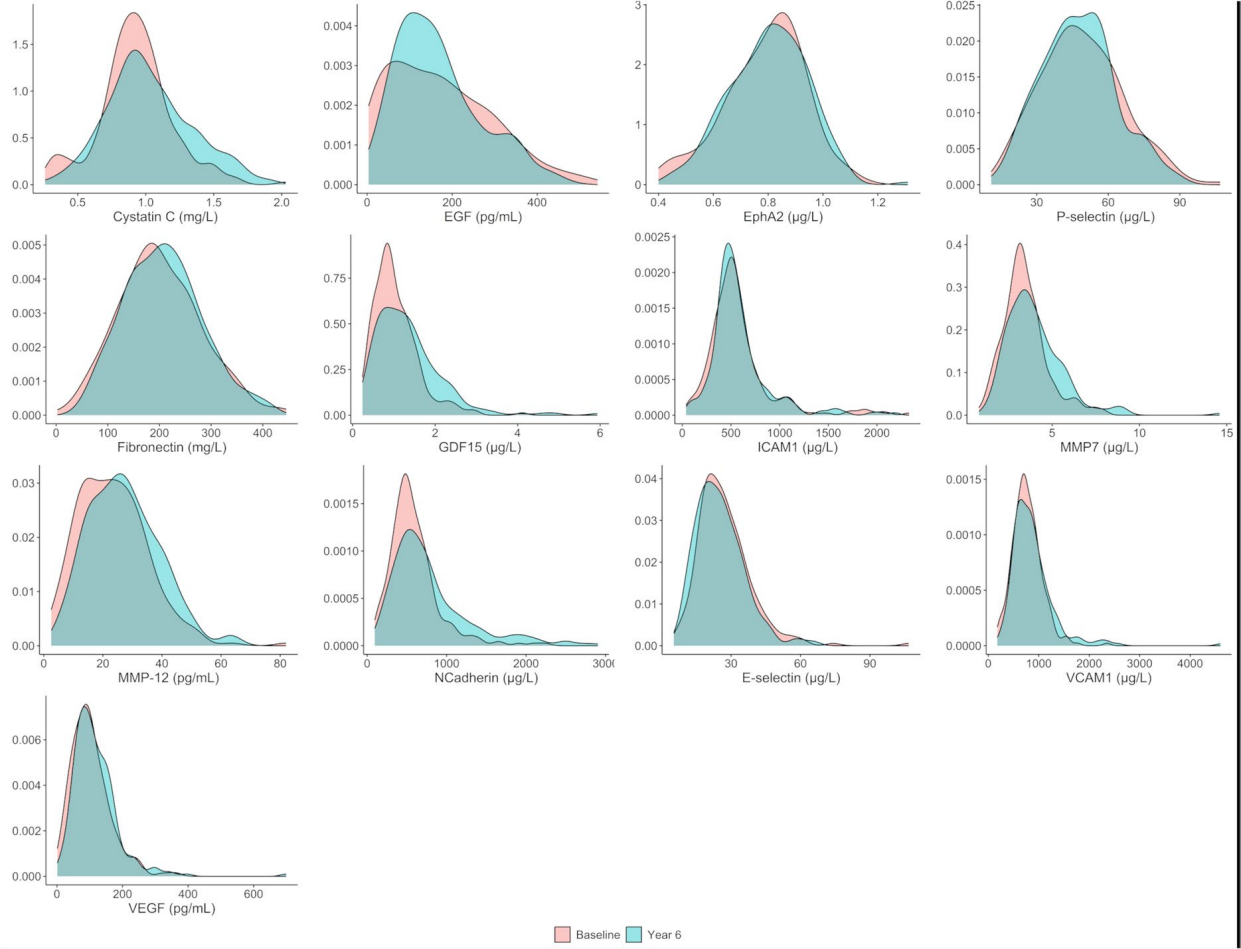
Density plots describing the distribution of vascular/organ dysfunction biomarker levels at baseline and at year six of follow-up. Abbreviations: EGF, epidermal growth factor; EphA2, ephrin type-A receptor 2; GDF-15, growth differentiation factor 15; ICAM-1, intercellular adhesion molecule 1; MMP, matrix metalloproteinase; VCAM-1, vascular cell adhesion molecule 1; VEGF, vascular endothelial growth factor

Figures 1, 2, 3, and 4 show the distribution of circulating concentrations of the 47 geroscience biomarkers at baseline and at follow-up across the four physiological biomarker domains analyzed. The mean serum concentrations and coefficient variation (%) of the biomarkers at baseline and follow-up are reported in Supplementary Table 1. The following biomarkers showed significantly higher concentrations at follow-up than at baseline: α-synuclein, adiponectin, β2M, C-peptide, CXCL10, cystatin C, EphA2, GDF-15, IGFBP-1, IL-6, IL-10, MMP7, MMP12, MPO, N-cadherin, renin, TNFRSF1B, VCAM- 1, and VEGF. E-selectin, IGFBP3, IL- 1α, IL- 1β, IL- 12p70, S100B, and Tau protein showed lower concentrations at year six than at baseline.

**Fig. 5:**
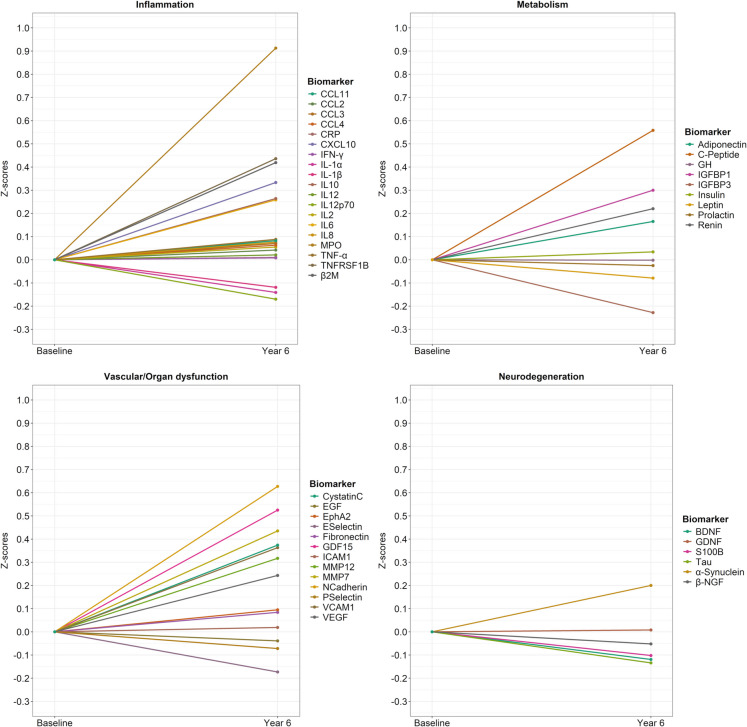
Biomarker *z*-score changes from baseline to year six using baseline values from the whole sample as the reference. Abbreviations: BDNF, brain-derived neurotrophic factor; β-NGF, *N*erve growth factor β; C-Peptide, connecting peptide; CCL, C-C motif chemokine ligand; CRP, C-reactive protein; CXCL10, C-X-C motif chemokine ligand 10; EGF, epidermal growth factor; EphA2, ephrin type-A receptor 2; GDF-15, growth differentiation factor 15; GDNF, glial cell line-derived neurotrophic factor; ICAM-1, intercellular adhesion molecule 1; IFN-γ, interferon gamma; IGFBP, insulin-like growth factor-binding protein; IL, interleukin; MMP, matrix metalloproteinase; MPO, myeloperoxidase; S100B, S100 calcium-binding protein B; TNF-α, tumor necrosis factor α; TNFRSF1B, tumor necrosis factor receptor superfamily member 1B; VCAM-1, vascular cell adhesion molecule 1; VEGF, vascular endothelial growth factor

The patterns of change in the mean *z*-score of each biomarker over the 6-year period are shown in Fig. 5. Different change patterns were identified across biomarkers. The major relative increase was observed for C-peptide, IGFBP1, MPO, TNFRSF1B, β2M, CXCL10, N-cadherin, GDF-15, MMP7, cystatin C, VCAM-1, and MMP12. The major decrease was observed for IGFBP2, IL-1α, IL-1β, IL-12p70, E-selectin, S100B, BDNF, and Tau protein. Relative changes in biomarker concentrations using participants in the youngest decade (60–69 years) as the reference group are depicted in Supplementary Fig. 1. The latter shows that, at baseline, most metabolic, inflammatory, and vascular/organ dysfunction and cellular senescence biomarkers had a mean *z*-score greater than 0, indicating that concentrations of those biomolecules were higher with increasing age. Patterns of change in biomarker concentrations over time were unaffected by the reference group.

### Correlations and clustering of biomarkers at baseline and follow-up

**Fig. 6:**
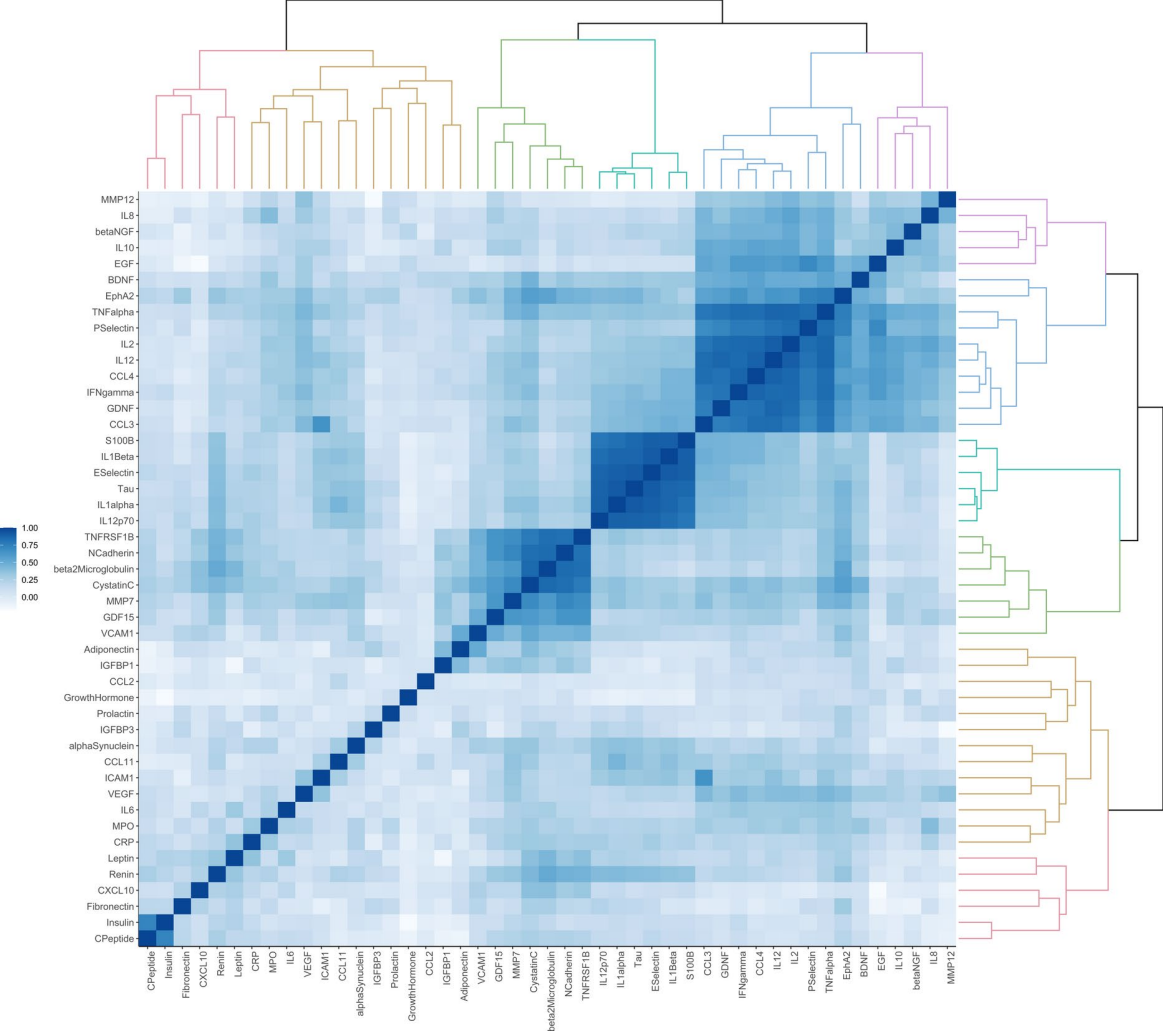
Correlation matrix and dendrogram of biomarkers at baseline. Abbreviations: BDNF, brain-derived neurotrophic factor; β-NGF, nerve growth factor β; C-Peptide, connecting peptide; CCL, C-C motif chemokine ligand; CRP, C-reactive protein; CXCL10, C-X-C motif chemokine ligand 10; EGF, epidermal growth factor; EphA2, ephrin type-A receptor 2; GDF- 15, growth differentiation factor 15; GDNF, glial cell line-derived neurotrophic factor; ICAM-1, intercellular adhesion molecule 1; IFN-γ, interferon gamma; IGFBP, insulin-like growth factor-binding protein; IL, interleukin; MMP, matrix metalloproteinase; MPO, myeloperoxidase; S100B, S100 calcium-binding protein B; TNF-α, tumor necrosis factor α; TNFRSF1B, tumor necrosis factor receptor superfamily member 1B; VCAM-1, vascular cell adhesion molecule 1; VEGF, vascular endothelial growth factor

**Fig. 7:**
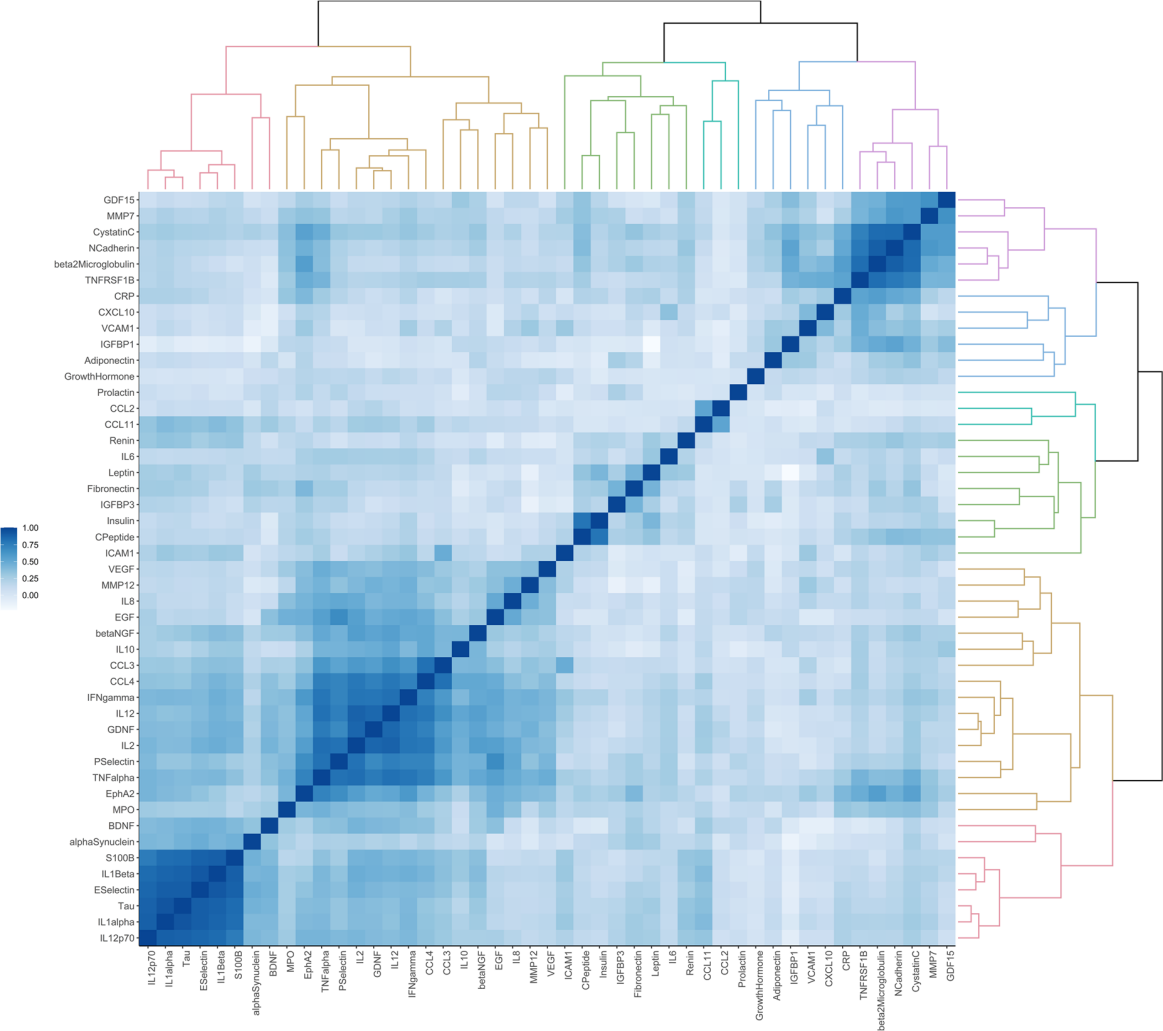
Correlation matrix and dendrogram of biomarkers at year six of follow-up. Abbreviations: BDNF, brain-derived neurotrophic factor; β-NGF, nerve growth factor β; C-Peptide, connecting peptide; CCL, C-C motif chemokine ligand; CRP, C-reactive protein; CXCL10, C-X-C motif chemokine ligand 10; EGF, epidermal growth factor; EphA2, ephrin type-A receptor 2; GDF-15, growth differentiation factor 15; GDNF, glial cell line-derived neurotrophic factor; ICAM-1, intercellular adhesion molecule 1; IFN-γ, interferon gamma; IGFBP, insulin-like growth factor-binding protein; IL, interleukin; MMP, matrix metalloproteinase; MPO, myeloperoxidase; S100B, S100 calcium-binding protein B; TNF-α, tumor necrosis factor α; TNFRSF1B, tumor necrosis factor receptor superfamily member 1B; VCAM-1, vascular cell adhesion molecule 1; VEGF, vascular endothelial growth factor

Correlations between pairs of biomarkers at baseline and at year six are depicted in Figs. 6 and 7, respectively. At both time points, six clusters of closely correlated biomarkers were identified based on their relative distance (Supplementary Table 2). Cluster composition differed between baseline and follow-up, except for the cluster including E-selectin, IL-1α, IL-1β, IL-12p70, S100B, and Tau protein, which remained stable.

### Association between age and longitudinal changes in biomarker levels

Coefficients and 95% confidence intervals of the association of age and sex with longitudinal changes in biomarker concentrations are reported in Table 3. A significant linear association between changes in age and biomarker concentrations over time was detected for the following biomolecules: (1) adiponectin, C-peptide, renin (metabolism), (2) CXCL10, IL-1α, IL-1β, IL-6, IL-10, IL-12p70, MPO (inflammation), (3) cystatin C, MMP7, MMP12, VCAM-1 (vascular/organ dysfunction and cellular senescence), and (4) S100B and Tau protein (neurodegeneration). Among these biomarkers, a negative association with increasing age was found with IL-1β, IL-1α, IL-12p70, S100B, and Tau protein.

**Table 3:** Association between age and longitudinal changes in biomarkers as assessed by linear mixed-effects models

**Biomarkers**	**Coefficients (95% confidence intervals)**		
	**Age**	**Sex (female)**	**Age**^**2**^
*Inflammation*			
β2-microglobulin	− 0.18 (− 0.39 to 0.03)	− 0.06 (0.50 to 0.38)	**0.002 (0.0004 to 0.003)**
CCL2	0.53 (− 1.83 to 2.88)	− 34.98 (− 112.87 to 42.91)	
CCL3	0.25 (− 0.54 to 1.04)	5.31 (− 14.64 to 25.25)	
CCL4	0.001 (− 0.001 to 0.004)	0.09 (0.02 to 0.16)	
CCL11	− 0.11 (− 1.20 to 0.98)	0.43 (− 27.05 to 27.92)	
CRP	0.02 (− 0.00 to 0.05)	0.06 (− 0.53 to 0.66)	
CXCL10	**0.67 (0.40 to 0.95)**	0.13 (− 6.55 to 6.81)	
IFNγ	0.07 (− 0.55 to 0.69)	12.75 (− 3.00 to 28.50)	
IL-1α	**− 0.22 (− 0.35 to − 0.10)**	0.73 (− 2.70 to 4.15)	
IL-1β	**− 0.16 (− 0.26 to –0.05)**	0.82 (− 2.13 to 3.78)	
IL-2	0.01 (− 0.07 to 0.10)	2.23 (0.13 to 4.33)	
IL-6	**0.12 (0.02 to 0.22)**	0.95 (− 1.49 to 3.39)	
IL-8	0.19 (− 0.02 to 0.39)	0.55 (− 4.15 to 5.25)	
IL-10	**0.06 (0.03 to 0.09)**	− 0.17 (− 0.87 to 0.52)	
IL-12	0.0005 (− 0.0024 to 0.0035)	0.07 (− 0.00 to 0.15)	
IL-12p70	**− 3.20 (− 4.81 to − 1.59)**	9.67 (− 35.63 to 54.97)	
MPO	**2.21 (1.22 to 3.21)**	− 7.20 (− 30.74 to 16.34)	
TNF-α	0.09 (− 0.00 to 0.17)	**2.39 (0.22 to 4.55)**	
TNFRSF1B	− 229.78 (− 469.72 to 10.15)	− 119.12 (− 614.29 to 376.06)	**2.07 (0.57 to 3.58)**
*Metabolism*			
Adiponectin	**0.14 (0.09 to 0.20)**	**2.46 (1.06 to 3.86)**	
C-peptide	**0.04 (0.02 to 0.05)**	**− 0.34 (− 0.65 to − 0.02)**	
Growth hormone	0.01 (− 0.00 to 0.02)	0.46 (0.14 to 0.78)	
IGFBP1	**− 5.58 (− 8.08 to − 3.09)**	3.05 (− 1.52 to 7.62)	**0.04 (0.02 to 0.06)**
IGFBP3	− 0.01 (− 0.01 to − 0.00)	0.14 (0.02 to 0.25)	
Insulin	1.04 (− 0.14 to 2.23)	− 17.27 (− 45.71 to 11.18)	
Leptin	**1.91 (0.78 to 3.03)**	**7.47 (4.84 to 10.11)**	**− 0.01 (− 0.02 to − 0.01)**
Prolactin	− 0.06 (− 0.29 to 0.17)	3.56 (− 2.50 to 9.62)	
Renin	**0.0053 (0.0015 to 0.0092)**	− 0.01 (− 0.10 to 0.09)	
*Neurodegeneration*			
α-synuclein	− 0.00 (− 0.00 to 0.00)	**0.07 (0.02 to 0.12)**	
BDNF	**1.64 (0.59 to 2.68)**	**2.39 (0.38 to 4.40)**	**− 0.01 (− 0.02 to − 0.00)**
GDNF	− 0.02 (− 0.09 to 0.05)	1.69 (− 0.12 to 3.50)	
NGF-β	0.00 (− 0.04 to 0.04)	0.36 (− 0.64 to 1.36)	
S100B	**− 2.17 (− 3.58 to − 0.76)**	3.82 (− 36.99 to 44.62)	
Tau protein	**− 1.33 (− 2.07 to − 0.60)**	7.13 (− 13.11 to 27.36)	
*Vascular/organ dysfunction and cellular senescence*			
Cystatin C	**0.01 (0.01 to 0.02)**	0.03 (− 0.04 to 0.10)	
EGF	− 0.57 (− 1.63 to 0.50)	**27.99 (2.98 to 53.00)**	
EphA2	**0.0026 (0.0012 to 0.0039)**	0.02 (− 0.01 to 0.06)	
E-selectin	− 0.23 (− 0.34 to − 0.13)	0.21 (− 2.72 to 3.15)	
Fibronectin	**9.31 (0.26 to 18.35)**	19.41 (− 0.13 to 38.95)	**− 0.06 (− 0.12 to − 0.00)**
GDF-15	**− 0.0769 (− 0.1506 to − 0.0033)**	**− 0.25 (− 0.40 to − 0.10)**	**0.0007 (0.0003 to 0.0012)**
ICAM-1	1.26 (− 1.11 to 3.63)	**− 97.24 (− 186.54 to − 7.95)**	
MMP12	**0.21 (0.10 to 0.33)**	− 2.01 (− 4.77 to 0.75)	
MMP7	**0.06 (0.04 to 0.07)**	− 0.07 (− 0.41 to 0.27)	
N-cadherin	**− 70.50 (− 118.67 to − 22.34)**	− 37.11 (− 130.79 to 56.57)	**0.58 (0.28 to 0.88)**
P-selectin	− 0.02 (− 0.18 to 0.14)	**6.43 (2.37 to 10.49)**	
VCAM-1	**12.41 (8.80 to 16.02)**	− 85.18 (− 174.68 to 4.31)	
VEGF	1.51 (0.87 to 2.15)	17.57 (0.55 to 34.59)	

Bold indicates statistically significant associations

*BDNF* brain-derived neurotrophic factor, *β-NGF* nerve growth factor β, *C-Peptide* connecting peptide, *CCL* C-C motif chemokine ligand, *CRP* C-reactive protein, *CXCL10* C-X-C motif chemokine ligand 10, *EGF* epidermal growth factor, *EphA2* ephrin type-A receptor 2, *GDF-15* growth differentiation factor 15, *GDNF* glial cell line-derived neurotrophic factor, *ICAM-1* intercellular adhesion molecule 1, *IFN-γ* interferon gamma, *IGFBP* insulin-like growth factor-binding protein, *IL* interleukin, *MMP* matrix metalloproteinase, *MPO* myeloperoxidase, *S100B* S100 calcium-binding protein B, *TNF-α* tumor necrosis factor α, *TNFRSF1B* tumor necrosis factor receptor superfamily member 1B, *VCAM-1* vascular cell adhesion molecule 1, *VEGF* vascular endothelial growth factor

Non-linear relationships, as indicated by a significant quadratic term of age, were found for the following biomarkers: IGFBP-1, leptin, β2M, fibrinogen, GDF-15, N-cadherin, TNFRSF1B, and BDNF.

## Discussion

The main results of our study revealed that repeated measures of 47 blood-borne biomarkers over 6 years showed differential associations with age. In particular, the following biomolecules were identified as the most relevant in the associations between age and repeated assessments of biomarker levels: (1) adiponectin, C-peptide, renin (metabolism); (2) CXCL10, IL-1α, IL-1β, IL-6, IL-10, IL-12p70, MPO (inflammation); (3) cystatin C, MMP7, MMP12, VCAM-1 (vascular/organ dysfunction and cellular senescence); and (4) S100B and Tau protein (neurodegeneration). Among these molecules, a negative association with increasing age was found for IL-1α, IL-1β, IL-12p70, S100B, and Tau protein.

Among all molecules analyzed, the identification of inflammatory and metabolic biomarkers as those that changed as a function of age is in line with a recent comprehensive analysis exploring multi-omics profiles during human aging [13]. In our study, adiponectin, C-peptide, and renin included in the metabolic domain showed a positive association with increasing age with concentrations that were higher at follow-up compared to baseline either before or after standardization to the baseline population. A concordant age-dependent increase over 6 years for these three biomarkers is in line with existing literature [14, 15]. The linear association of these markers with age may indicate a role for these molecules as metabolic markers of aging and/or biomarkers of metabolic syndrome in older populations [16]. Adiponectin, a hormone also referred to as adipokine and mainly produced by adipose tissue but also muscle and brain, regulates glucose levels and fatty acid breakdown. Adiponectin enhances insulin sensitivity and prevents atherosclerosis [17, 18]. High circulating levels of adiponectin are associated with decreased risk of developing type 2 diabetes in middle aged individuals [14] and low adiponectin levels are reduced in states of insulin resistance (i.e., obesity and type 2 diabetes) [19, 20]. However, in older adults, high adiponectin levels are associated with muscle loss, greater risk of incident disability, and mortality [21, 22]. Similarly, high levels of the connecting peptide, C-peptide, of the insulin A-chain and B-chain in the proinsulin molecule have been found to be associated with body composition and diabetes in older adults [23, 24]. C-peptide is an indicator of bodily insulin production and a marker of glucose homeostasis, also associated with telomere shortening [15], likely indicating that metabolic dysregulation may have a role in premature aging. High circulating levels of adiponectin have also been reported as predictors of incident cardiovascular and renal events in treated hypertensive patients [25] and to be released upon blockade of the renin-angiotensin system in patients with essential hypertension to improve insulin sensitivity [26]. Renin, an enzyme that regulates blood volume and pressure, has been shown to be decreased in its level and activity with aging [27]. However, levels of this enzyme can also be elevated in old age because of dehydration that is frequent in older adults, who typically have a lower volume of water in their bodies and/or may have conditions and/or take medications that increase the dehydration risk. In this setting, the increase in serum levels of renin may be envisioned as a marker of hypovolemia.

These metabolic regulators, and especially adiponectin, can also exert anti-inflammatory, antioxidant, and anti-apoptotic functions, with significant inverse correlation with pro-inflammatory cytokines [28]. In keeping with this is the longitudinal age-related decrease of IL-1α, IL-1β, and IL-12p70 at follow-up compared to baseline either before or after standardization to the baseline population of our study. Of note, this subset of mediators belongs to the only cluster of analytes that, together with S100B and Tau protein, did not show any difference in composition between baseline and follow-up (Figs. 6 and 7). S100B and Tau protein are biomarkers primarily associated with brain health and neurological conditions [29, 30]. S100B is a protein released by astrocytes and is often used as a marker of brain injury or neuroinflammation. It has been linked to conditions like traumatic brain injury, stroke, and neurodegenerative diseases [30, 31]. The finding of low levels of S100B with age is in line with existing literature showing that S100B tends to decrease slightly with age, and low levels of S100B in older adults may be an indicator of non-significant brain injury or inflammation. Tau protein is a stabilizer of microtubules in neurons. In certain neurodegenerative conditions, such as Alzheimer’s disease, Tau becomes hyperphosphorylated and forms tangles, which disrupt normal brain function. An increase in circulating Tau protein is strongly associated with neurodegenerative diseases or traumatic brain injury [32–36]. Low levels of circulating Tau in older adults, instead, may indicate less neurodegeneration or ongoing brain damage [37].

A longitudinal age-related increase in CXCL10, IL-6, IL-10, and MPO was identified and paralleled by greater concentrations of markers of vascular/organ dysfunction and cellular senescence (i.e., cystatin C, MMP7, MMP12, and VCAM-1). An increased circulating concentration of MMP7 and MMP12 may indicate active extracellular matrix remodeling that is involved in degrading the elastic layers and/or cell membranes basement with promotion of initiation and progression of atherosclerosis. MMP12 is a macrophage-produced metalloelastase that supports elastin degradation in the extracellular matrix and may also enable immune cell infiltration and inflammation. IL-6 and IL-10 are among the inflammatory mediators released by macrophages that may infiltrate the extracellular environment. Such a transition may be also assisted by an elevated expression of the cell adhesion glycoprotein VCAM-1 in endothelial cells. The age-related increase in this subset of pro-inflammatory mediators accompanies the development of a state of chronic low-grade inflammatory in older adults that predisposes to the development and progression of frailty [37, 38]. An age-related interplay between inflammatory signals and the development of vascular/organ dysfunction and cellular senescence has been associated with more specific conditions such as cardiovascular aging [39–43]. In line with this are also the high levels of cystatin C, an inhibitor of cysteine proteases at the extracellular level, that is removed from the bloodstream by the kidneys. Higher circulating levels of this protein have been reported in older adults with greater cardiovascular risk and are a better estimator of glomerular filtration than creatinine [44, 45]. Furthermore, increased cystatin C levels are associated with frailty and mortality [46, 47].

Finally, non-linear relationships were identified with age for IGFBP-1, leptin, β2M, TNFRSF1B, fibrinogen, GDF-15, N-cadherin, and BDNF. This finding is in line with recent research demonstrating the existence of nonlinear relationship between blood-borne biomarkers of aging and the development of chronic diseases across the human lifespan [13]. GDF-15 was also associated with mortality risk in men older than 60 [48]. Moreover, a hormetic role was proposed for GDF-15 in skeletal muscle [49]. Beneficial effects have been attributed to GDF-15 when expressed at high levels in muscles facing acute stress. Conversely, maladaptive effects (e.g., low-grade inflammation) were attributed to GDF-15 in chronic conditions (e.g., obesity, aging) [49]. Similarly, fibrinogen was reported to reflect the frailty status at the cross-sectional level while not associated with frailty progression over an 8-year period [50]. On a different note, the soluble TNFRSF1B has recently been shown to have greater reproducibility compared with IL-6 in the assessment of chronic inflammation in older adults to the point that it was proposed as a novel inflammatory marker of aging [51].

From a clinical perspective, adiponectin, cystatin C, and GDF-15 may be suitable for inclusion in aging biomarker panels or risk scores. Adiponectin, which regulates insulin sensitivity and inflammation, could help identify individuals at higher risk for metabolic syndrome or atherosclerosis. Cystatin C, given its association with kidney function, systemic inflammation, and frailty, may be included in multimarker panels to assess biological age and predict mortality risk independent of traditional risk factors. GDF-15, a cytokine associated with inflammation and cellular stress and linked to frailty, cognitive decline, and cardiovascular disease, when incorporated into aging panels could provide valuable insights into overall health status and predict adverse outcomes like heart failure or mortality.

Some limitations of our study need to be acknowledged. First, the study was based on a relatively small random selection of a larger sample population. While most characteristics of the SNAC-K population were reliably represented, additional associations might have been detected by analyzing a larger sample subset. Second, the sample population was limited to individuals residing in the Kungsholmen district of Stockholm, with most participants being of Caucasian descent. Therefore, findings may not be generalizable to other ethnic groups or regions. Third, by selecting individuals with valid assessments at both baseline and 6-year follow-up, we necessarily excluded those who died during the first 6 years of the study, potentially introducing a survival selection bias, which makes our results more applicable to healthier older adults. Fourth, due to sample availability, biomarkers were measured in serum, which can affect their stability as compared with plasma. Some biomarkers may exhibit higher degradation in serum due to clotting, which could interfere with measurement accuracy. However, most serum biomarkers assayed have shown a good concordance with their plasma values [52, 53]. Furthermore, most previous studies also assayed biomarkers in serum. Lastly, while our biomarker selection was based on extensive literature evidence, we missed other relevant biomarkers of aging that were not measurable due to unavailability of commercial kits for the analytical platform used. Future studies including a larger set of biomolecules and enrolling more heterogeneous populations are warranted to investigate more in-depth age-dependent biomarker variation over time.

## Conclusion

The analysis of repeated measures of blood-borne biomarkers allowed identification of inflammatory and metabolic biomolecules as those more strongly associated with aging over six years of follow-up. Nonlinear relationships were also identified. While providing insights on some of the events underlying the aging process and leading to negative health-related events, a more comprehensive multi-omics longitudinal analysis in larger cohorts is needed to capture the nuances that allow understanding the complex of aging.

## Supplementary Information

Below is the link to the electronic supplementary material.

Supplementary file1 (DOCX 143 KB)

## Data Availability

The data employed in the present study are available upon request at https://www.snac-k.se/ and with the approval of the SNAC-K Principal Investigator and Steering Group.

## Abbreviations

B2M: β2-microglobulin
BDNF: Brain-derived neurotrophic factor
β-NGF: Nerve growth factor β
C-Peptide: Connecting peptide
CCL: C-C motif chemokine ligand
COPD: Chronic obstructive pulmonary disease
CRP: C-reactive protein
CXCL10: C-X-C motif chemokine ligand 10
EGF: Epidermal growth factor
EphA2: Ephrin type-A receptor 2
GDF- 15: Growth differentiation factor 15
GDNF: Glial cell line-derived neurotrophic factor
ICAM- 1: Intercellular adhesion molecule 1
IFN-gamma: Interferon gamma
IGFBP: Insulin-like growth factor-binding protein
IL: Interleukin
MMP: Matrix metalloproteinase
MPO: Myeloperoxidase
S100B: S100 calcium-binding protein B
SNAC-K: Swedish National Study on Aging and Care in Kungsholmen
SD: Standard deviation
TNF: Tumor necrosis factor
TNFRSF1B: Tumor necrosis factor receptor superfamily member 1B
VCAM- 1: Vascular cell adhesion molecule 1
VEGF: Vascular endothelial growth factor
