# Supplementary material for: Longitudinal changes in blood-borne geroscience biomarkers: results from a population-based study
Source: GeroScience. 2025 Apr 24;47(5):6411–27. doi: 10.1007/s11357-025-01666-x (PMC12634922; doi:10.1007/s11357-025-01666-x)
Supplement: Supplementary file 1 — Supplementary file1 (DOCX 143 KB) [file 11357_2025_1666_MOESM1_ESM.docx]

**Table S1.** Mean levels and coefficient variation of biomarkers at baseline and follow-up.

| **Biomarkers** | **Baseline (N=234)** | **Follow-up (N=234)** | **p-value**  paired t-test | **Biomarkers** | **Baseline (N=234)** | **Follow-up (N=234)** | **p-value**  paired t-test |
| --- | --- | --- | --- | --- | --- | --- | --- |
| **Adiponectin** |  |  |  | **CCL2** |  |  |  |
| Mean (SD) | 11.4 (5.3) | 12.2 (6.1) | **0.002** | Mean (SD) | 416 (299) | 428 (275) | 0.186 |
| CV% | 47.0% | 49.8% |  | CV% | 72.1% | 64.2% |  |
| **Renin** |  |  |  | **CCL3** |  |  |  |
| Mean (SD) | 0.48 (0.31) | 0.55 (0.45) | **0.005** | Mean (SD) | 550 (74) | 555 (85) | 0.275 |
| CV% | 65.6% | 82.9% |  | CV% | 13.5% | 15.3% |  |
| **Insulin** |  |  |  | **CCL4** |  |  |  |
| Mean (SD) | 149 (117) | 153 (125) | 0.639 | Mean (SD) | 1.59 (0.26) | 1.61 (0.27) | 0.288 |
| CV% | 78.6% | 81.7% |  | CV% | 16.4% | 17.0% |  |
| **C-Peptide** |  |  |  | **MPO** |  |  |  |
| Mean (SD) | 2.01 (1.19) | 2.67 (1.49) | **<0.001** | Mean (SD) | 154 (550) | 189 (112) | 0.335 |
| CV% | 59.3% | 55.8% |  | CV% | 356.9% | 59.3% |  |
| **Prolactin** |  |  |  | **Fibronectin** |  |  |  |
| Mean (SD) | 33.7 (22.9) | 33.2 (24.2) | 0.632 | Mean (SD) | 201 (80) | 208 (75) | 0.119 |
| CV% | 67.7% | 72.9% |  | CV% | 39.8% | 36.2% |  |
| **IGFBP-1** |  |  |  | **E-Selectin** |  |  |  |
| Mean (SD) | 19.1 (19.1) | 24.8 (23.5) | **<0.001** | Mean (SD) | 27.3 (11.9) | 25.2 (10.4) | **<0.001** |
| CV% | 100.1% | 94.7% |  | CV% | 43.5% | 41.3% |  |
| **IGFBP-3** |  |  |  | **MMP-7** |  |  |  |
| Mean (SD) | 1.08 (0.51) | 0.96 (0.40) | **<0.001** | Mean (SD) | 3.44 (1.27) | 3.99 (1.67) | **<0.001** |
| CV% | 47.0% | 41.9% |  | CV% | 36.8% | 41.8% |  |
| **Growth hormone** |  |  |  | **MMP-12** |  |  |  |
| Mean (SD) | 1.41 (1.72) | 1.41 (1.46) | 0.983 | Mean (SD) | 23.4 (11.9) | 27.2 (11.9) | **<0.001** |
| CV% | 121.7% | 103.5% |  | CV% | 50.6% | 43.8% |  |
| **Leptin** |  |  |  | **EGF** |  |  |  |
| Mean (SD) | 14.1 (10.9) | 13.3 (10.8) | 0.104 | Mean (SD) | 172 (122) | 167 (100) | 0.575 |
| CV% | 77.1% | 81.6% |  | CV% | 70.8% | 59.6% |  |
| **CRP** |  |  |  | **VEGF** |  |  |  |
| Mean (SD) | 2.73 (2.51) | 2.75 (2.54) | 0.886 | Mean (SD) | 103 (61) | 118 (73) | **<0.001** |
| CV% | 92.1% | 92.2% |  | CV% | 59.0% | 61.9% |  |
| β**2-Microglobulin** |  |  |  | **P-Selectin** |  |  |  |
| Mean (SD) | 3.20 (1.63) | 3.88 (2.20) | **<0.001** | Mean (SD) | 48.8 (17.2) | 47.6 (15.6) | 0.188 |
| CV% | 50.9% | 56.6% |  | CV% | 35.3% | 32.8% |  |
| **CXCL10** |  |  |  | **EphA2** |  |  |  |
| Mean (SD) | 35.8 (21.6) | 43.0 (33.7) | **<0.001** | Mean (SD) | 0.79 (0.15) | 0.80 (0.14) | **0.047** |
| CV% | 60.5% | 78.5% |  | CV% | 18.9% | 17.7% |  |
| **IL-1**β |  |  |  | **VCAM-1** |  |  |  |
| Mean (SD) | 36.8 (11.4) | 35.4 (11.1) | **0.009** | Mean (SD) | 769 (292) | 876 (453) | **<0.001** |
| CV% | 31.1% | 31.5% |  | CV% | 38.0% | 51.7% |  |
| **IL-1**α |  |  |  | **ICAM-1** |  |  |  |
| Mean (SD) | 46.6 (13.7) | 44.7 (12.7) | **0.002** | Mean (SD) | 579 (346) | 586 (311) | 0.453 |
| CV% | 29.3% | 28.5% |  | CV% | 59.7% | 53.1% |  |
| **IL-12 p70** |  |  |  | **GDF-15** |  |  |  |
| Mean (SD) | 562 (177) | 532 (168) | **<0.001** | Mean (SD) | 1.05 (0.55) | 1.34 (0.80) | **<0.001** |
| CV% | 31.6% | 31.6% |  | CV% | 52.8% | 59.9% |  |
| **TNFRSF1B** |  |  |  | **N-Cadherin** |  |  |  |
| Mean (SD) | 3760 (1790) | 4540 (2500) | **<0.001** | Mean (SD) | 590 (321) | 791 (508) | **<0.001** |
| CV% | 47.6% | 55.1% |  | CV% | 54.5% | 64.2% |  |
| **CCL11** |  |  |  | **Cystatin C** |  |  |  |
| Mean (SD) | 218 (106) | 226 (115) | 0.194 | Mean (SD) | 0.93 (0.27) | 1.03 (0.31) | **<0.001** |
| CV% | 48.6% | 50.8% |  | CV% | 29.4% | 30.6% |  |
| **TNF-α** |  |  |  | **Tau** |  |  |  |
| Mean (SD) | 38.1 (8.9) | 38.8 (8.8) | 0.148 | Mean (SD) | 276 (80) | 265 (77) | **0.004** |
| CV% | 23.3% | 22.7% |  | CV% | 28.9% | 28.9% |  |
| **IL-2** |  |  |  | **S100B** |  |  |  |
| Mean (SD) | 34.3 (8.2) | 34.7 (9.2) | 0.422 | Mean (SD) | 532 (157) | 516 (153) | **0.015** |
| CV% | 23.8% | 26.4% |  | CV% | 29.5% | 29.6% |  |
| **IL-6** |  |  |  | α**-Synuclein** |  |  |  |
| Mean (SD) | 11.2 (6.4) | 12.8 (12.6) | **0.009** | Mean (SD) | 1.01 (0.27) | 1.07 (0.23) | **0.012** |
| CV% | 57.5% | 97.7% |  | CV% | 26.9% | 21.6% |  |
| **IL-8** |  |  |  | β-**NGF** |  |  |  |
| Mean (SD) | 38.1 (24.9) | 40.0 (18.9) | 0.313 | Mean (SD) | 11.9 (4.1) | 13.2 (17.0) | 0.647 |
| CV% | 65.4% | 47.3% |  | CV% | 34.7% | 128.7% |  |
| **IL-10** |  |  |  | **GDNF** |  |  |  |
| Mean (SD) | 4.39 (2.52) | 5.05 (2.91) | **<0.001** | Mean (SD) | 28.6 (7.2) | 28.7 (7.3) | 0.886 |
| CV% | 57.5% | 57.6% |  | CV% | 25.2% | 25.3% |  |
| **IL-12** |  |  |  | **BDNF** |  |  |  |
| Mean (SD) | 1.51 (0.31) | 1.52 (0.30) | 0.716 | Mean (SD) | 23.9 (8.6) | 22.9 (8.2) | 0.063 |
| CV% | 20.3% | 19.6% |  | CV% | 35.8% | 35.8% |  |
| **IFN-γ** |  |  |  |  |  |  |  |
| Mean (SD) | 274 (63) | 274 (64) | 0.887 |  |  |  |  |
| CV% | 22.9% | 23.3% |  |  |  |  |  |

Abbreviations: BDNF, Brain-derived neurotrophic factor; β-NGF, Nerve growth factor β; C-Peptide, Connecting peptide; CCL, C-C motif chemokine ligand; CRP, C-reactive protein; CXCL10, C-X-C motif chemokine ligand 10; EGF, Epidermal growth factor; EphA2, Ephrin type-A receptor 2; GDF-15, Growth differentiation factor 15; GDNF, Glial cell line-derived neurotrophic factor; ICAM-1, Intercellular adhesion molecule 1; IFN-γ, Interferon gamma; IGFBP, Insulin-like growth factor-binding protein; IL, Interleukin; MMP, Matrix metalloproteinase; MPO, Myeloperoxidase; S100B, S100 calcium-binding protein B; TNF-α, Tumor necrosis factor; TNFRSF1B, Tumor necrosis factor receptor superfamily member 1B; VCAM-1, Vascular cell adhesion molecule 1; VEGF, Vascular endothelial growth factor; CV, coefficient of variation; SD, Standard deviation

**Table S2.** Biomarker clusters at baseline and follow-up.

| **Cluster** | **Baseline** | **Follow-up** |
| --- | --- | --- |
| 1 | C-peptide, insulin, fibrinogen, CXCL10, renin, leptin | ICAM1, C-peptide, insulin, IGFBP3, fibrinogen, leptin, IL-6, renin |
| 2 | CRP, MPO, IL-6, VEGF, ICAM1, CCL11, α-Synuclein, IGFBP3, prolactin, growth hormone, CCL2, IGFBP1, adiponectin | CCL11, CCL2, prolactin |
| 3 | VCAM1, GDF15, MMP7, cystatin C, β2-microglobulin, N-cadherin, TNFRSF1B | TNFRSF1B, β2-microglobulin, N-cadherin, cystatin C, MMP7, GDF15 |
| 4 | IL12p70, IL1α, Tau, E-selectin, IL-1β, S100B | IL12p70, IL1α, Tau, E-selectin, IL-1β, S100B, α-Synuclein, BDNF |
| 5 | CCL3, GDNF, IFN-gamma, CCL4, IL12, IL2, P-selectin, TNF-α, EphA2, BDNF | MPO, EphA2, TNF-α, P-selectin, IL12, IL2, GDNF, IFN-gamma, CCL3, CCL4, IL-10, β-NGF, EGF, IL-8, MMP12, VEGF |
| 6 | EGF, IL-10, β-NGF, IL-8, MMP12 | Growth hormone, adiponectin, IGFBP1, VCAM1, CXCL10, CRP |

Abbreviations: BDNF, Brain-derived neurotrophic factor; β-NGF, Nerve growth factor β; C-Peptide, Connecting peptide; CCL, C-C motif chemokine ligand; CRP, C-reactive protein; CXCL10, C-X-C motif chemokine ligand 10; EGF, Epidermal growth factor; EphA2, Ephrin type-A receptor 2; GDF-15, Growth differentiation factor 15; GDNF, Glial cell line-derived neurotrophic factor; ICAM-1, Intercellular adhesion molecule 1; IFN-γ, Interferon gamma; IGFBP, Insulin-like growth factor-binding protein; IL, Interleukin; MMP, Matrix metalloproteinase; MPO, Myeloperoxidase; S100B, S100 calcium-binding protein B; TNF-α, Tumor necrosis factor; TNFRSF1B, Tumor necrosis factor receptor superfamily member 1B; VCAM-1, Vascular cell adhesion molecule 1; VEGF, Vascular endothelial growth factor

**Figure S1.** Z-score standardization and changes of biomarkers over a 6-year period with baseline measures of participants aged 60−69 years as the reference group.


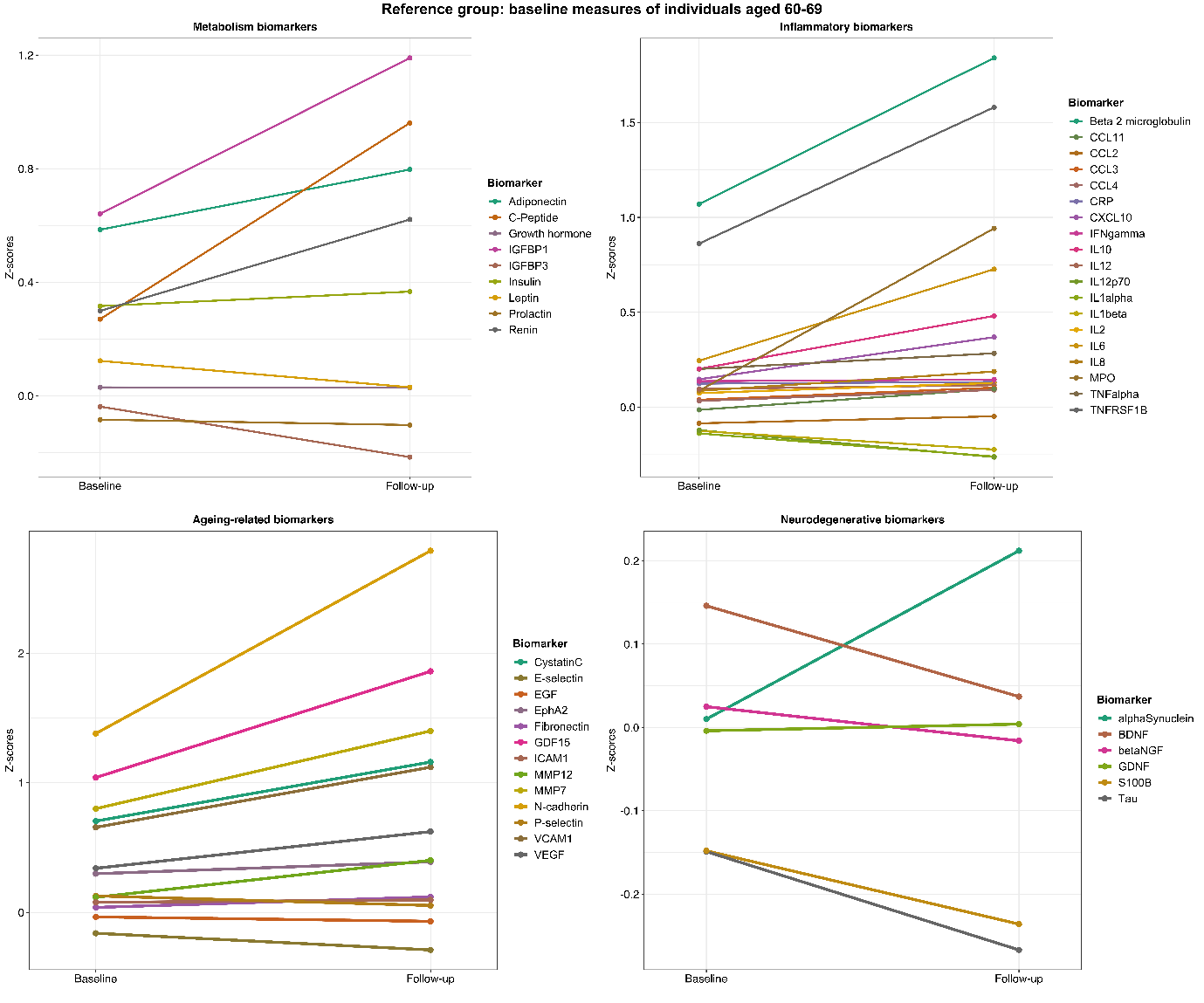


Abbreviations: BDNF, Brain-derived neurotrophic factor; β-NGF, Nerve growth factor β; C-Peptide, Connecting peptide; CCL, C-C motif chemokine ligand; CRP, C-reactive protein; CXCL10, C-X-C motif chemokine ligand 10; EGF, Epidermal growth factor; EphA2, Ephrin type-A receptor 2; GDF-15, Growth differentiation factor 15; GDNF, Glial cell line-derived neurotrophic factor; ICAM-1, Intercellular adhesion molecule 1; IFN-γ, Interferon gamma; IGFBP, Insulin-like growth factor-binding protein; IL, Interleukin; MMP, Matrix metalloproteinase; MPO, Myeloperoxidase; S100B, S100 calcium-binding protein B; TNF-α, Tumor necrosis factor; TNFRSF1B, Tumor necrosis factor receptor superfamily member 1B; VCAM-1, Vascular cell adhesion molecule 1; VEGF, Vascular endothelial growth factor
